# Doctoral training to support sustainable soil geochemistry research in Africa

**DOI:** 10.1098/rsfs.2023.0058

**Published:** 2024-08-09

**Authors:** M. G. Manzeke-Kangara, I. S. Ligowe, B. Kaninga, P. Nalivata, V. Kabambe, E. Mbewe, B. H. Chishala, G. M. Sakala, P. Mapfumo, F. Mtambanengwe, T. Tendayi, A. Murwira, A. D. C. Chilimba, F. P. Phiri, E. L. Ander, E. H. Bailey, R. M. Lark, K. Millar, M. J. Watts, S. D. Young, M. R. Broadley

**Affiliations:** ^1^ Rothamsted Research, West Common, Harpenden, UK; ^2^ Department of Soil Science and Environment, University of Zimbabwe, Harare, Zimbabwe; ^3^ School of Biosciences, University of Nottingham, Sutton Bonington, UK; ^4^ Department of Crop and Soil Sciences, Lilongwe University of Agriculture and Natural Resources, Lilongwe, Malawi; ^5^ Department of Agricultural Research Services, Lilongwe, Malawi; ^6^ Department of Forestry and Environmental Management, Mzuzu University, Mzuzu, Malawi; ^7^ Zambia Agriculture Research Institute, Mount Makulu, Central Research Station, Lusaka, Zambia; ^8^ School of Agricultural Sciences, University of Zambia, Great East Road Campus, Lusaka, Zambia; ^9^ Department of Geography, Geospatial Sciences and Earth Observation, University of Zimbabwe, Harare, Zimbabwe; ^10^ Department of Nutrition, HIV and AIDS, Ministry of Health, Lilongwe, Malawi; ^11^ Inorganic Geochemistry, Centre for Environmental Geochemistry, British Geological Survey, Nottinghamshire, UK

**Keywords:** doctoral training networks, laboratory investments, PhD 'thinkers', research partnerships, strengthened research capacity

## Abstract

Africa’s potential for scientific research is not yet being realized, for various reasons including a lack of researchers in many fields and insufficient funding. Strengthened research capacity through doctoral training programmes in higher education institutes (HEIs) in Africa, to include collaboration with national, regional and international research institutions, can facilitate self-reliant and sustainable research to support socio-economic development. In 2012, the Royal Society and the UK’s Department for International Development (now the Foreign, Commonwealth and Development Office) launched the Africa Capacity Building Initiative (ACBI) Doctoral Training Network which aimed to strengthen research capacity and training across sub-Saharan Africa. The ACBI supported 30 core PhD scholarships, all registered/supervised within African HEIs with advisory support from the UK-based institutes. Our ‘Soil geochemistry to inform agriculture and health policies’ consortium project, which was part of the ACBI doctoral training programme network, was implemented in Malawi, Zambia and Zimbabwe between 2014 and 2020. The aims of our consortium were to explore linkages between soil geochemistry, agriculture and public health for increased crop productivity, nutrition and safety of food systems and support wider training and research activities in soil science. Highlights from our consortium included: (i) the generation of new scientific evidence on linkages between soils, crops and human nutrition; (ii) securing new projects to translate science into policy and practice; and (iii) maintaining sustainable collaborative learning across the consortium. Our consortium delivered high-quality science outputs and secured new research and doctoral training funding from a variety of sources to ensure the continuation of research and training activities. For example, follow-on Global Challenges Research Funded Translation Award provided a strong evidence base on the prevalence of deficiencies in children under 5 years of age and women of reproductive age in Zimbabwe. This new evidence will contribute towards the design and implementation of a nationally representative micronutrient survey as an integral part of the Zimbabwe Demographic and Health Surveys conducted by the Ministry of Health and Child Care. The award also generated new evidence and a road map for creating quality innovative doctorates through a doctoral training landscape activity led by the Zimbabwe Council for Higher Education. Although our project and the wider ACBI has contributed to increasing the self-reliance and sustainability of research within the region, many challenges remain and ongoing investment is required.

## 1. Introduction

Doctoral training opportunities continue to increase globally, to inform national development, knowledge economy and improve the quality of universities through first-class teaching and learning experience [1,2]. The global view of doctoral training as the foundation for a knowledge-based economy has led to some countries, e.g. South Africa’s National Development Plan, setting a target of achieving 5000 doctorates per year by 2030 from 1876 in 2012 [2], while others, e.g. China [3,4] and the USA [5], are already surpassing these numbers. For example, Cyranoski *et al.* [3] reported that 50 000 students graduated with doctorates across all disciplines in China in 2009 alone. However, enrolment and graduation from doctoral training in Africa remain limited, largely due to a lack of capacity and/or poorly structured PhD programmes [2]. A lack of embedded research capacity has often left institutions unaware of how best to support PhD students.

Addressing global scientific imbalances helps the economies and public welfare of individual countries while increasing diversity in global scientific research [6]. Some Governments, through their education ministries, have launched national development programmes to address this challenge. For example, the Ministry of Higher and Tertiary Education, Innovation, Science and Technology Development of Zimbabwe launched the country’s Education 5.0 policy in 2021, which extended the higher education institutions’ (HEIs’) mission of teaching, research and community service, to include innovation and industrialization [7]. African-based initiatives such as the Regional Universities Forum for Capacity Building in Agriculture (RUFORUM) have also supported national universities and their respective ministries of education to build and strengthen the capacity of academic and research institutions.

This article highlights the importance of doctoral training and presents evidence from one such programme working on doctoral training, the Africa Capacity Building Initiative (ACBI), funded by the UK’s Department for International Development (DfID; now the Foreign, Commonwealth and Development Office, FCDO) and the Royal Society (RS) [8]. We highlight project objectives from our ‘Soil geochemistry to inform agriculture and health policies’ consortium project, which was one of 10 consortia within the ACBI programme. Our consortium sought to address challenges in dietary micronutrient supplies, and potentially harmful elements, emanating from soil geochemical processes linked to agricultural practices. The consortium project, conducted in Malawi, Zambia and Zimbabwe was designed to address existing skills gaps in soil geochemistry, including analytical chemistry, experimental design, geospatial data collection and integration, geochemical and statistical modelling, soil management and generic presentation, communication and grant writing skills in these three countries. We present scientific highlights and outcomes from our consortium including individual PhD students’ successes, capacity building in technical support, research ethics, wider societal and environmental benefits emanating from the project and linkages with industry and new projects to support the translation of the research into policy and practice, and to stimulate new science. We conclude by presenting enablers and barriers towards sustainable research and a need for a paradigm shift towards a thriving and sustainable knowledge economy in Africa.

## 2. The Africa Capacity Building Initiative

In 2012, the RS and the FCDO (formerly DfID) launched the ACBI, aimed at strengthening research capacity and training across sub-Saharan Africa (SSA) by creating new research networks. The ACBI supported 30 core PhD scholarships across 10 projects in the areas of soil science, water and sanitation for health (WASH) and renewable energy [8]. A key feature of the ACBI was that all core PhD students were registered and supervised primarily within African universities, i.e. HEIs, with advisory support from both African-based research institutes and the UK-based HEIs and research institutes. In addition to delivering high-quality research through PhD training, the consortia were also tasked with improving laboratory and technical capacity and supporting the development of sustainable research partnerships, including ‘south–south’ and ‘south–north’ links, to support future African-led research.

The ACBI was guided by the following objectives:

1. To facilitate sustainable multidisciplinary partnerships between research groups in SSA and the UK in the areas of soil science, WASH and renewable energy.
2. To strengthen research and training capacity in SSA HEIs through south–south, south–north and north–south collaborations, including skill transfer between partner organizations of the research consortia.
3. To produce a cadre of early career, talented researchers through integrated PhD scholarships and shared supervision of postgraduate students.
4. To examine the nature of skill gap patterns within the research consortia and to identify the major barriers and enablers at the institutional level in providing cost-efficient and high-quality post-graduate training.

During the initial phase of the ACBI (2013–2014), 20 networking grants were awarded. These networking grants enabled new partnerships to be developed. The ACBI went on to support 10 full research consortia and 30 core PhD scholarships. Each consortium was awarded up to £1.2 million over 5 years period. After a competitive selection process [9], the first-round awards began in 2015 and the second-round awards started in 2016. The award covered PhD and technical training and research costs, a small budget for equipment and travel and subsistence costs. A well-defined monitoring, evaluation and learning (MEL) component was delivered by independent external experts at the Centre for Capacity Research (CCR, formerly the Capacity Research Unit, CRU) at the Liverpool School of Tropical Medicine (LSTM) [8,9]. The MEL component aimed to determine capacity strengths and gaps in postgraduate training and research systems, identify common strengths and gaps across institutions, present programme reports, peer-reviewed papers, expert commentary and provide feedback to the RS–FCDO to guide in-country programme improvements.

## 3. Consortium project ‘Soil geochemistry to inform agriculture and health policies’

The overall aim of our project was to support training and research in soil science in three countries in southern Africa: Malawi, Zambia and Zimbabwe. Our science focus was on developing a greater understanding of linkages between soil geochemistry, agriculture and public health, to support improvements in the productivity, nutritional quality and safety of food systems. We sought to generate evidence to support policy decisions relating to nutrient management in agricultural systems, and public health interventions in areas at risk of micronutrient deficiencies and toxicities. For example, specific science questions were asked regarding the bioavailability of micronutrients and their influence on crop production and human nutrition across multiple spatial scales. The research included the use of predictive models derived from mechanistic understanding of soil–plant transfers, geospatial analyses and soil management strategies. The consortium’s research work was informed by earlier collaborative studies within and between some of the consortium partners [10–12], although the consortium had not all worked together previously.

Our consortium trained core and aligned PhD students, laboratory scientists and researchers from local government institutions and universities within each country. In general, the African researchers had substantial experience in understanding tropical smallholder cropping systems and soil science, with UK researchers having experience in soil science and statistics, alongside access to technologies in soil and plant analytical techniques that were not available in Malawi, Zambia and Zimbabwe. Through the consortium, we were able to invest in laboratory equipment and training of laboratory scientists. The consortium also provided strong support in training in research ethics, data management and academic writing through annual meetings and regional and international exchange visits.

Interactions with the policy sector were embedded within our network. Each partner in Malawi, Zambia and Zimbabwe was represented by both HEI and the main government research body with a remit on soil science/agriculture. In Malawi, this body is the Department of Agricultural Research Services (DARS) within the Ministry of Agriculture, Irrigation and Water Development. In Zambia, the body is the Zambia Agriculture Research Institute (ZARI) within the Ministry of Agriculture and Livestock. In Zimbabwe, it is the Chemistry and Soil Research Institute (CSRI) of the Department of Research and Specialist Services (DR&SS) within the Ministry of Lands, Agriculture, Water, Fisheries & Rural Development.

Senior staff from each of these government bodies contributed to: (i) PhD supervision and mentorship; (ii) training of other students and technical staff; and (iii) providing access to facilities and resources. All of these organizations contributed towards network-wide training activities in years 1–6 of our project. Our model ensured that all research and development and training had an ultimate focus on policy relevance and potential policy outcomes.

### 3.1. Core PhD training

Three (core-funded) PhD students drove the research activities, supported by technical staff and laboratory capacity strengthening activities. Academics within the consortium also supported further (i.e. aligned, non-core-funded) studentships within the network. The core-funded students were registered at universities in Malawi (Ivy Sichinga Ligowe), Zambia (Belinda Kaninga) and Zimbabwe (Muneta Grace Manzeke). Each of their projects embedded three training themes: (i) soil geochemistry; (ii) geospatial analyses; and (iii) soil management aimed at answering the main research questions detailed in table 1. The following section provides a background to each project and some of the research highlights.

**Table 1.** Summary on core students’ research focus, methods and core findings.

core student	main research questions	methods used	core findings/highlights
Ivy Ligowe	does soil type affect iodine and selenium crop uptake and fixation?	soil and foliar selenium and iodine biofortification to green vegetables (*Amaranthus* and *Brasca*)	soil iodine and selenium and foliar iodine biofortification to green vegetables has the potential to increase dietary iodine and selenium supply
		laboratory analysis of green vegetables and soils	selenium and iodine biofortification to green vegetables subjected to repeated harvests would require repeated biofortification to consistently enhance both selenium and iodine concentrations in the harvested leaves
		statistical linear mixed modelling	soil-applied selenium is quickly fixed in the soil and is not available for plant uptake after three weeks of biofortification
			soil acidity control measures should be considered in acidic soils to enhance soil selenium and iodine plant uptake
	does organic input to the soil through soil management (conservation agriculture, CA) affect the crop uptake and soil fixation of selenium (isotopic Se^77^) and their residual effect on the following crop?	selenium agronomic biofortification field studies	organic input in CA cropping systems has no negative influence on the uptake and fixation of the agronomically biofortified Se^77^. The soil-applied Se^77^ is not available beyond the year it is applied; yearly application is recommended
		laboratory analysis of grain, biomass and soils	
		statistical linear mixed modelling	
Belinda Kaninga	do crops grown on soils that are contaminated by heavy metals take up harmful concentrations of the heavy metals?	on-farm field and greenhouse pot trials	soil-to-plant transfer of heavy metals in contaminated soil is evident but depends on the type of crop. For example, pumpkin leaves tend to accumulate high concentrations of heavy metals compared with maize grain
	do lime and manure affect the soil-to-plant transfer of heavy metals?	laboratory soil analysis methods	lime and manure can reduce the soil-to-plant transfer of heavy metals
		geospatial analysis	
		statistical linear mixed modelling	
Grace Kangara	do geospatial variations in soil zinc and iron concentration and improved farmer soil fertility management strategies influence grain zinc and iron nutritional quality of cereals and grain legumes constituting diets of smallholder communities in Zimbabwe?	qualitative and quantitative survey tools including participatory action research, farmer focus group discussions and household questionnaires	micronutrient distribution in soils and grains is governed by various factors including soil type, field productivity level and crop type
		on-farm field trials	integrated soil fertility management practices and micronutrient fertilizer application improve crop productivity and grain quality
		laboratory analysis methods	
		statistical linear mixed modelling	

### 3.2. Ivy S. Ligowe: the biogeochemical dynamics of iodine and selenium in contrasting soil types in Malawi

**Figure 1.**
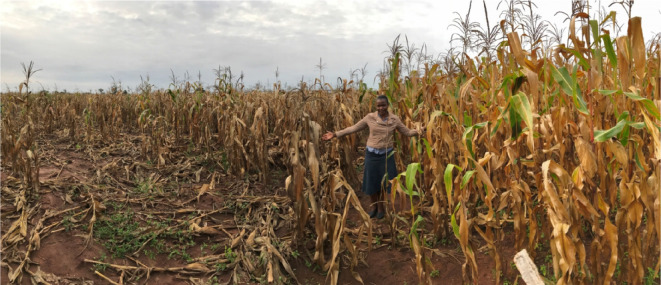
Dr Ivy Ligowe showing differences between conventional (*left*) and CA (*right*) treatment plots at Chitedze Research Station, DARS, Malawi on 25 April 2017.

Ivy’s project aimed to understand the mechanisms governing selenium and iodine dynamics in tropical soils and to develop a geospatial tool to optimize their management in soils and crops. Selenium and iodine are micronutrients that are essential for the health of people and grazing livestock, and deficiencies of both elements are widespread in Malawi and many other countries in SSA [13–16]. Ivy’s studies were centred around a long-term (> 10 years) field experiment, at Chitedze in Central Malawi (figure 1), consisting of contrasting soil organic management treatments and their effects on corn/maize (*Zea mays* L.) production and quality. This experiment had been designed originally to test the principles of conservation agriculture (CA) on crop system productivity, including reduced tillage, crop rotations and retention of organic materials [17]. Studies at this site included the application of selenium fertilizers which had been shown to be a feasible method to improve selenium in the edible portions of maize crops in Malawi [11]. Soils at this site (Alfisol), and from two further sites with Oxisol and Vertisol soils, in Dedza and Ngabu-Chikwawa, respectively, were also used for controlled-environment studies. These three soil types are important in Malawi and occur widely in SSA. A novel aspect of Ivy’s project was the use of stable isotopes to trace micronutrient fluxes in different systems.

One of Ivy’s research highlights was to show the effects of selenium fertilizers on crop quality, under different field conditions and different farmer practices, and the fate of the selenium in the environment [18,19]. Ivy reported the rapid fixation of fertilizer-applied selenium with organic matter in the soil (‘humus-bound’ selenium) which means that selenium fertilizers are largely unavailable to plants beyond the growing season in which the fertilizer is applied. Therefore, selenium fertilizers would need to be applied annually to improve the grain quality of crops in Malawi. Potential negative impacts of selenium build-up in the soil were looked at by Mathers *et al.* [20] during implementation of field trials under three contrasting soil types in the UK. Findings from their study, which calculated losses due to leaching and selenium build-up over time demonstrated that doubling soil selenium concentrations would take about 500 years with annual selenium fertilizer application. Walsh & Fleming [21] reported small environmental consequences from yearly applications of 20 g selenium fertilizer ha^−1^, requiring more than 3000 years to reach toxicity levels of 20 mg kg^−1^. Annual selenium fertilizer applications and incorporation of selenium-enriched crop residues are unlikely to result in selenium toxicity [22]. For example, little effect of CA treatments (e.g. annual retention of crop residues) on selenium uptake by maize under control or selenium-fertilized conditions was observed in Malawi [19]. However, some studies reported negative correlations between soil pH and soil selenium content [23,24]. This implies management practices such as liming and organic nutrient resource use, which increase soil pH, should be employed under predominantly acid soils in SSA, to reduce the possibility of long-term soil selenium toxicity. Additional papers from Ivy’s work are in table 2.

**Table 2.** Peer-reviewed publications which acknowledge funding from ACBI project AQ140000.

Lark *et al*. [25]. https://doi.org/10.5194/soil-3-235-2017
Lark *et al*. [26]. https://doi.org/10.1111/ejss.12449
Manzeke *et al*. [27]. ISBN:978−0−85310−431−5
Manzeke *et al*. [28]. https://doi.org/10.1016/j.fcr.2017.08.010
Manzeke *et al*. [29]. https://doi.org/10.1038/s41598-019-42828-0
Kaninga *et al*. [30]. https://doi.org/10.1007/s10653-019-00326-2
Phiri *et al*. [15]. https://doi.org/10.1038/s41598-019-43013-z
Phiri *et al*. [31]. https://doi.org/10.1016/j.envint.2019.105218
Phiri *et al*. [32]. https://doi.org/10.1007/s10653-020-00700-5
Manzeke *et al*. [33]. https://doi.org/10.1002/agj2.20175
Ligowe *et al*. [18]. https://doi.org/10.1016/j.geoderma.2019.114106
Ligowe *et al*. [19]. https://doi.org/10.1016/j.geoderma.2020.114315
Ligowe *et al*. [34]. https://doi.org/10.1017/S0029665120006904
Ligowe *et al*. [16]. https://doi.org/10.1007/s10653-020-00714-z
Kaninga *et al*. [35]. https://doi.org/10.1016/j.heliyon.2020.e05502
Hamilton *et al*. [36]. https://doi.org/10.1016/j.chemosphere.2020.125984
Gashu *et al*. [37]. https://doi.org/10.1038/s41586-021-03559-3
Manzeke-Kangara *et al*. [38]. https://doi.org/10.1186/s43170-021-00057-4
Manzeke-Kangara *et al*. [39]. https://doi.org/10.3390/agronomy11010124
Kaninga *et al*. [40]. https://doi.org/10.1007/s10653-021-00849-7
Botoman *et al*. [41]. https://doi.org/10.1038/s41598-022-12014-w
Kumssa *et al*. [42]. https://doi.org/10.1038/s41597-022-01500-5

### 3.3. Belinda Kaninga: bioavailability of heavy metals in soils that are in close proximity to mine tailings in Copperbelt, Zambia

Belinda’s project aimed to understand how soil management affects the phyto-availability of heavy metal contaminants in agricultural soils arising from mining activities in Zambia. Heavy metal contaminants in agricultural soils can contaminate food crops through plant uptake and via resuspension of soil dust, and can cause adverse health effects [43,44]. Field sites in proximity to Mugala village and Luanshya mine tailings, both located in the Copperbelt region of northern Zambia were characterized. Field experimental work was done to determine the influence of liming and manure application on soil-to-crop transfer of heavy metal contaminants, including novel stable isotope analyses to understand how soil management affects the lability of heavy metals and their uptake into crops. Belinda explored the use of the Windermere Humic Aqueous Model to predict heavy metals fractionation, solubility and speciation in tropical soils affected by mine tailings and geostatistics to assess geospatial variation in heavy metals bioavailability in soils near mine tailings.

One of Belinda’s research highlights was to increase our understanding of the soil-to-plant transfer of several heavy metals, including cadmium, copper, lead and nickel, by maize crops and pumpkin leaves, when these crops are grown in soils amended with lime and manure, and the downstream risks of dietary exposure to the local community [40]. The concentrations of heavy metals in maize grain were not affected by the soil amendments. The heavy metal concentrations in maize grain were below the prescribed FAO/WHO safe limits for all metals. However, the concentration of the heavy metals was reduced in pumpkin leaves when grown in soils amended with lime and manure. There was evidence that pumpkin leaves are likely to remain a greater exposure route for contaminants into human diets. Links to Belinda’s additional papers are in table 2.

### 3.4. M. Grace Kangara: geospatial variation of bioavailable micronutrients in tropical soils and its effects on crop productivity and human nutrition

Grace’s project aimed to understand factors affecting the bioavailability of zinc and iron in tropical soils, and how this affects crop productivity and human nutrition. Zinc and iron are micronutrients that are essential for the health of people and grazing livestock and for maximizing crop yields for farmers. The work included studies on smallholder farms in two districts of Zimbabwe (Hwedza and Mutasa), representing contrasting agro-ecological zones, alongside work at a range of field sites. Field surveys were conducted using a nested sampling design to determine factors governing zinc and iron supply in soils and crops grown under different farmer soil fertility management practices and soil types. Field experimental work was conducted to determine the effects of zinc and iron fertilizer application and different nitrogen fertilizer application forms (i.e. mineral, organic or co-applications thereof) on grain micronutrient concentration of maize, different finger millet landraces and cowpea. Fertilizer treatments and crop type choices were selected to suit different farmer management practices.

One of Grace’s research highlights was to show that integrated soil fertility management (i.e. involving combinations of chemical and organic fertilizers, including the use of cattle manures and leaf litter) can improve the yield and micronutrient quality of a range of cereal and legume crops [29]. The widespread adoption of these approaches could improve profits in the farming sector and reduce the prevalence of micronutrient deficiencies in Zimbabwe and the wider region [38]. Links to Grace’s additional papers are in table 2.

### 3.5. Aligned PhD training

In addition to core-funded PhD students, we recruited several additional ‘aligned’ PhD students registered in both African-based and UK-based institutions. For example, Felix Phiri’s research highlights included the first comprehensive, nationally representative, survey of the selenium status of a population in Malawi [15,31]. Felix showed there was widespread and severe selenium deficiency at a national scale in Malawi with strong evidence of geospatial clustering of selenium status as had been predicted previously based on soil and food system characteristics [10,14,45,46]. Felix published a literature review of selenium deficiency in Malawi food systems as a co-first author with Ivy Ligowe, supporting cross-disciplinary interactions. Felix’s PhD was funded by the University of Nottingham and the British Geological Survey, with substantial ‘in kind’ contributions from the Government of Malawi and their implementing partners who were undertaking the National Micronutrient Survey of 2015/2016. This work has since supported many policy interactions and the co-development of policy briefs with the Government of Malawi, through subsequent GeoNutrition and Micronutrient Action Policy Support (MAPS) projects, funded by the Bill & Melinda Gates Foundation (BMGF).

Elliott Hamilton’s work explored the mechanistic controls on the transfer of hexavalent chromium [(Cr(VI)] from soil to crops using novel elemental speciation and isotope dilution methods. Elliott worked with Belinda Kaninga at the same field sites in the Copperbelt Province, with funding from the British Geological Survey. The distribution of elemental species of chromium in soils near mine tailings potentially pose significant human health challenges. Elliott’s work reported a low environmental/human health risk at an agricultural site near mine tailings in the Copperbelt Province of Zambia based on significantly lower concentrations of Cr(VI) of between 0.03 and 0.29 mg kg^−1^ compared to a residential UK screening value for Cr(VI) of 21 mg kg^−1^ [36]. Aligned students attended training activities and helped to support longer-term network sustainability through their contributions and stimulated ongoing research and development linkages with the UK and wider academic engagement.

### 3.6. Wider laboratory and technical development

There have been investments in laboratory equipment and training of laboratory scientists as part of the laboratory and technical development initiative within the ACBI’s Soil Geochemistry consortium. A microwave plasma-atomic emission spectroscopy was installed in each of the lead institutions in the three African countries. Technical staff aligned with the consortium (Emmanuel Mbewe, Takesure Tendayi) attended project meetings and visited the UK as part of the technical capacity strengthening activity of the project. Technicians were involved in follow-on projects and networks which is key in sustainable research. For example, Takesure is an advisor and trainer for the FAO-UN Global Soil laboratory network—GLOSOLAN (https://www.fao.org/global-soil-partnership/glosolan/en/), a network aimed at investing in global soil laboratories; demonstrating the wider benefits of the consortium. Research is highly dependent on the technical infrastructure, including personnel, particularly for PhD students who are highly dependent on technical support during their first years of study. Technical staff provide the potential for longevity in capacity development.

### 3.7. Bibliometric analyses of publications

**Figure 2.**
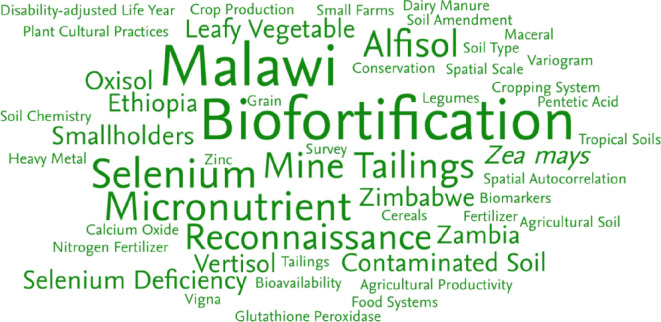
Top 50 keywords generated by SciVal based on relevance from the 21 ACBI publications.

In total, 21 peer-reviewed papers have been published to date by the consortium, which attribute ACBI funding, either as primary outputs, led by core students, or as part of other projects (table 2). Figure 2 shows the top 50 keywords/phrases by relevance, based on 21 publications, as generated by SciVal. These 21 publications represent contributions from a total of 50 different authors, based at 18 different institutes worldwide; 11 of these institutes are based in an African country. Most (17/21) of the outputs have a first author based at an African institution. While article bibliometrics should not be considered as a substitute for peer assessment of quality, they can provide useful summary information around the academic ‘reach’ of the research. For example, a SciVal summary (conducted on 3 November 2023) reveals that these 21 papers had been cited 452 times: a mean of 21.5 citations per paper. The Field-Weighted Citation Impact was 1.87, which indicates that these ACBI publications had been cited 87% more times (i.e. almost double) than the mean number of citations received by all other publications globally in the same subject fields.

## 4. Follow-on projects to support sustainability of research and development activities

Our ACBI project facilitated further funding proposals which were developed and submitted by members of the consortium, including with new partners.

Successful larger projects included the GeoNutrition projects (2017–2023) funded primarily by the BMGF and the UKRI’s Global Challenges Research Fund (GCRF). The primary geographical focus was Ethiopia and Malawi including a leading role for Lilongwe University of Agriculture and Natural Resources (LUANAR). More than 10 new PhD studentships were registered through GeoNutrition, mostly in Ethiopia and Malawi, building directly on our ACBI consortium model. Outputs of GeoNutrition included primary papers [37,41,42,47–50] and co-developed policy briefings for policymakers, which included socio-economic and ethical analyses of agricultural policies and practices to alleviate micronutrient malnutrition. The GeoNutrition project itself then led to further BMGF funding via the ‘Micronutrient Action Policy Support (MAPS) Tool’ project (2019–2024). The MAPS project, which is the modelling of dietary micronutrients under current and future scenarios, has a wider pan-Africa focus than GeoNutrition. MAPS has released an open-access web-hosted decision support tool for use by policymakers in agriculture, nutrition and health sectors, by the private sector and by others including research donors, the education sector and NGOs [51,52].

A GCRF Research Translation Award ‘Translating GeoNutrition’ was secured to extend the work into Zimbabwe, with a leading role for the University of Zimbabwe (UZ), with support from Malawi, again, with new studentships registered at UZ. Findings from this work provided a strong evidence base on the prevalence of deficiencies in children under 5 years of age and women of reproductive age [53–55]. This new evidence will contribute to the design and implementation of a nationally representative micronutrient survey to be conducted in the next Zimbabwe Demographic and Health Survey by the Ministry of Health and Child Care. The Translation Award also generated new evidence and a road map for creating quality innovative doctorates from a doctoral training landscape activity led by the Zimbabwe Council for Higher Education [7]. This work will inform policies and transform micronutrient initiatives and the education landscape in Zimbabwe.

A successful bid was made to the GCRF Collective Fund GROW programme on research capacity strengthening. The project, CEPHaS, was focussed on the impacts of CA practices on the physical properties of soils, and implications for soil water storage, transfers to groundwater and the overall impact on water and food security. Capacity strengthening was focussed on the relevant physical sciences, including soil physics, hydrogeology and geophysical methods. There was also substantial training in statistics including sampling and experimental design. The project team included researchers from the original ACBI team, but also a wider range of disciplines from within the institutions (e.g. soil physicists and hydrogeologists) and additional partner institutions (Rothamsted Research and the CCR from LSTM). This project represented a substantial investment in research capacity strengthening to support decisions on soil and groundwater management. Progress was somewhat hindered by the COVID-19 pandemic, but this also testified to the robustness of the capacity developed in African institutions, where staff were able to trouble-shoot and maintain field instrumentation on the basis of training received in the project when UK-based staff could not travel to assist. Members of the CEPHaS team reflected on the process by which the project was put together, building on collaborative relationships developed between individuals and institutions through ACBI, in response to an article on how to tackle the phenomenon of ‘helicopter research’ in soil science [56]. Helicopter research refers to the phenomenon of researchers from resource-rich countries appearing in SSA countries to conduct research with no genuine partnership with local institutions and researchers. The CEPHaS project placed a strong emphasis on the development of early career researchers and gave them hands-on experience of project management and leadership.

Outputs from the CEPHaS project to date included a review and assessment of statistical functions for the prediction of soil hydraulic properties [57], and a paper on the implications of yield variability in a long-term CA experiment for the design of future experiments [58]. This led to further work in the context of GeoNutrition to address the problem of under-powered field experiments [59], which has been extended to the problem of design of on-farm trials in the context of CGIAR’s Excellence in Agronomy programme. New links were established with the fertilizer sector and DARS in Malawi to support new field trials to guide the formulation of new micronutrient fertilizer blends to boost micronutrient nutrition in these systems. This work is a follow-on of studies conducted by Botoman *et al.* [59,60] and will provide information, required by fertilizer industry and policy sector on: (i) maize grain yield increases due to zinc fertilizer application, the main benefit driving zinc fertilizer adoption by farmers; (ii) optimal zinc rates for agronomic zinc biofortification of maize in different agro-ecological zones; and (iii) cost–benefit analyses and potential business/regulatory models. The primary intended outcome will be approved recommendations supported by industry and governmental partners, to include acceptable business and regulatory models for the use of zinc fertilizers for health. Ultimate impacts of this translation project will be reduced zinc deficiency and improved public health.

The CEPHaS team successfully bid for funding in related areas of soil science including the evaluation of legacy soil information from colonial- and post-colonial soil surveys, in collaboration with historians in UNZA and the University of Nottingham (funded by the UKRI’s Arts and Humanities Research Council) [61,62]. A successful bid was also made to the European Joint Programme for Soils (CropGas project) to extend research on CEPHaS sites to examine the impacts of CA on greenhouse gas emissions from soil. This project also involves partners in Ireland, Poland and South Africa as well as the UK, widening further the international collaboration and networks of the partners in Zambia, Zimbabwe and Malawi.

## 5. Reflections of the consortium

The notion that PhD graduates must be problem solvers and not just specialists [63] cannot be overemphasized, especially in SSA where the need for accelerated economic development is key. The World Bank recommends that universities should train more researchers in response to societal challenges posed by climate change, disease, food insecurity and political instability [64]. This means that PhD training must equip one with key competencies to conduct impactful research. These skills include critical thinking, experimental, computational, research ethics, leadership and communication skills, among others.

However, most PhD programmes in universities in the developing world follow the traditional unstructured apprenticeship type of training, which may not effectively equip a candidate with the necessary industry-driven skills [65]. The ACBI offered the PhD students training opportunities in communication skills, and, more specifically, in our Soil Geochemistry consortium, experimental skills, statistics, GIS, research ethics and integrity and data management. The multidisciplinary nature of the wider consortium and its partners gave the students an opportunity to a wider resource base, e.g. supervisory expertise and equipment. For example, stable isotope and ion speciation studies by Belinda and Ivy would have been difficult without access to specialized spectroscopic equipment which was not available at their local institutions. The ACBI project engaged with lead laboratory technicians, emphasizing the importance of collaborative and multidisciplinary approaches to solving problems in the research process. Furthermore, the collaboration of research institutions and universities ensured that the PhD projects were not just an academic exercise but aligned with national research priorities.

**Figure 3.**
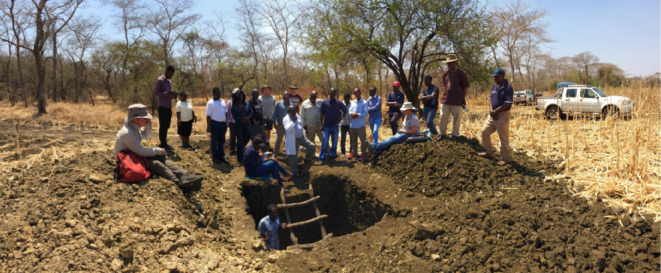
Professor Maxon Lowole (in white coat) giving a lecture on soil profiles in the Vertisol soil pit at DARS, Chitala Malawi on 10 September 2016.

For the supervisory team, the consortium served as a platform for collaboration, knowledge sharing and networking. For example, during annual meetings which were held rotationally among the member countries, local experts in the key thematic project areas worked with project experts to offer training (see figure 3). Training participants would include non-consortium students and members of staff of the hosting institutions. Thus, the training and networking benefits of the consortium can be extended to the wider membership of the participating institutions.

The Soil Geochemistry consortium advanced micronutrients research in African food systems and how their intake and toxicities thereof could be managed in these communities. Implementation of policies that ensure research advances to wider populations through mandated use of micronutrient fertilizer in these agri-based production systems and mechanisms to monitor subsequent concentrations within the wider soil–crop–food–feed–livestock–human nexus are therefore imperative.

## 6. Enablers and barriers towards self-reliant and sustainable research

### 6.1. Evidence from the soil geochemistry consortium

The Soil Geochemistry consortium under the ACBI doctoral training programme is a successful example of a programme that can contribute towards a self-reliant and sustainable research landscape in Africa. We highlight here some of the enablers and barriers encountered within our consortium, spanning from the initial PhD registration processes to the integration of academic and technical support staff (table 3). While the evidence base is from one consortium, it can inform the scope and structure of future wider collaborative research programmes.

**Table 3.** Evidence from the Soil Geochemistry consortium on enablers and barriers that facilitated or hindered the collaboration, communication and supervision of students.

category	enablers	barriers
PhD registration	clear project proposal outline submitted during grant application stage. This allowed students to have a clear understanding of their project and how they could contribute to the overall project. This preliminary understanding of the project shortened the time for student proposal development and submission to the academic approval board	the complexity of the overall project potentially influenced students to focus on overall consortium goals rather than specific projects
laboratory facilities	students and technical staff had access to modern laboratory equipment. New analytical instruments were installed at each lead partner institution for establishing new activities	students and technical support staff had to familiarize themselves with new equipment and quality assurance such as use of certified reference materials, delays in procurement/repairs
project meetings and trainings	project meetings enabled students to establish links and networks. Training received in research ethics, analytical chemistry, experimental design, geospatial data collection and integration, geochemical and statistical modelling, soil management, and generic presentation, communication and grant writing skills	transferability of newly acquired skills could be challenging. Ongoing mentorship ensured this did not impede the implementation and successful completion of the project
student supervision and progress reporting	all supervisors had training in research supervision. Regular student progress reports offered an opportunity to review what was going well and what needed to be addressed	students encountered minor delays due to the COVID-19 pandemic. For example, there were no guidelines for online PhD defence, and these had to be developed to support students
integration of academic and technical support staff	links were strengthened between academic and technical support staff through project meetings and training	challenging to secure time for personal development and training in addition to technical roles in the laboratories
scientific publication of work and examination	at least two published manuscripts were mandatory before PhD thesis submission at all host universities. This equipped students with technical writing skills and ensured the quality of work, including the thesis, through academic peer-review process	long review processes for papers delayed the submission of thesis for examination
peer-to-peer student interactions	good peer-to-peer interactions through project meetings and emails. This provided a strong support structure for motivation and learning	internalizing another student’s work to provide wider contexts during presentation proved challenging.

Elaborated from overall ACBI assessments presented by Refs. [8,9] to present experiences from the Soil Geochemistry consortium.

Networking is one of the core benefits gained during the ACBI initiative: the Soil Geochemistry consortium has opened doors for networking both internationally and locally with academic institutions, Government departments and CGIAR centres. Using the network platforms developed during the ACBI programme, I can partner in new research projects. Skills gained during the ACBI doctoral training include: writing skills, presentation skills, experimental design, data collection, statistical data analysis using R, grant writing, project management skills gained through the involvement in big projects like the Bill & Melinda Gates Foundation-GeoNutrition and CEPHaS (UKRI GCRF) projects. No major pronounced challenges were experienced during the doctoral training.—Dr Ivy Ligowe, Mzuzu University.

The multidisciplinary nature of the project team gave me a good opportunity to conduct a well-balanced study. Having specialists, within the team, in core aspects of a geochemistry/scientific research such as experimental layout, laboratory analysis, data management, statistical analysis meant that attention would be given to all these areas. My PhD training experience enhanced my research career as I am now more critical in my research approaches.—Dr Belinda Kaninga, ZARI.

I received high quality scientific guidance during my PhD from my supervisors and mentors. The tremendous amount of learning opportunities was incredible. I gained scientific knowledge on varying areas relevant to research. The one I cherish the most is on ethics. I am now more conscious of research ethical considerations when implementing my projects.—Dr M. Grace Kangara, Rothamsted Research.

### 6.2. Global imbalances in scientific research capacity

The limited number of researchers in certain institutions and disciplines, including the natural and applied sciences, continues to hamper efforts towards self-reliant and sustainable research in SSA. Despite a steady increase in number of children attending school since 1970 [66], the share of the population with completed tertiary education has remained below 5% [64]. This is further reflected by lower number of active researchers per million inhabitants between 7 and 437 in 2016 in SSA compared with over 4000 researchers per million inhabitants in developed countries [67,68]. In addition, most studies in developing countries focus on applied rather than basic research [69]. While applied problems may be most pressing, the development of a healthy scientific system with scope for future innovation also requires that active development of fundamental understanding is also progressing, and that scientists with an applied focus have access to collaborators who can bring new ideas to bear [70].

### 6.3. Example strategies for addressing scientific research imbalances

Research is an integral component for socio-economic development. While there is potential for world-class pioneering research in SSA, there are many challenges, including fewer researchers in selected fields, a lack of resources (e.g. funding, equipment and access to international networks) and competing donor priorities [71]. National research strategies are important in support of research and are often set in line with current global challenges and agendas. For example, the Government of Zimbabwe introduced the Zimbabwe Agenda for Socio-Economic Transformation (ZimASSET; 2013–2018), which aimed to provide sustainable economic empowerment and social transformation for its nationals. The ZimASSET economic blueprint coincided with the development of the UN’s Agenda 2030 on Sustainable Development Goals (SDGs) which have a global call to action to end poverty, protect the planet and ensure peace and prosperity [72] and the African Union’s Agenda 2063 [73].

Although most African countries have strategic research agendas, diversion from set objectives, including for entirely pragmatic reasons/externalities, can impede the self-reliance and sustainability of most research for development initiatives. Taking ownership by setting the research agenda and determining research priorities by African governments ensures that research serves the societal and economic developmental needs of local people [74], who ultimately own the right and responsibility to use the evidence. Although it is not yet possible for research to be funded entirely at a national level, it is essential that external support seeks to be aligned with the government strategic objectives.

Malawi is among the SSA countries that could benefit hugely from increased investment in research capacity. In 2010, overall enrolment in tertiary education was among the lowest in SSA where only about 1% of Malawi’s qualified cohort were enrolled in tertiary education [75]. There have been substantial ongoing investments in HEIs in Malawi. For example, LUANAR, formerly Bunda College of Agriculture, had five faculties by 2016 enrolling approx. 3750 undergraduates with approx. 40% of them being female. Despite having 77 postgraduate students (65 taught and 12 PhDs) in 2016, there were no PhD students enrolled in the Faculty of Agriculture or the Faculty of Food and Human Sciences in 2015. Given the many pressing challenges in food security, this is an issue that clearly needs to be addressed to align with the countries’ strategic goals for development.

By 2054, there will be a marked increase in the working-age population in most African countries including Malawi [76,77]. However, development challenges such as the economic burden of the youthful population in Malawi requires a change in the structure of the country’s population for the youthful dependency burden to be reduced and subsequent economic benefits from the working age to be realized [78]. In Malawi, this demographic dividend is largely due to a reduction in the total fertility rate, average birth rate and dependency burden on the working group [77]. The employment gap in Malawi will rise from 2.5 to 13.4 million between 2014 and 2054 [79]. This large labour force with fewer dependent children is likely to result in accelerated economic growth within a pessimistic environment of availability of job opportunities. Within the context of these projected changes, governments should seek to accelerate the socio-economic transformation and economic growth by strengthening the capacity of doctoral students and creating national centres of excellence in research to help ensure the full realization of the dividends arising from demographic changes.

### 6.4. Recognition of research outputs ownership and partnerships

The ownership of research outputs and/or the right and responsibility to use research evidence generated using official development assistance (ODA) budgets remains controversial. Inequality in the proportion of grants invested between donor and African countries often leads to power asymmetries, and subsequently to a lack of commitment and effort by African researchers. While it is often impossible to separate politics and research, it is fundamental that external funding focuses on strengthening the research and development capacity of countries they seek to assist. By empowering local researchers, local policymakers will be able to balance evidence-based findings more effectively, and therefore make appropriate and relevant decisions for their countries.

## 7. The plight of African doctoral students: an urgent need for paradigm shift

### 7.1. Core pillars for sustainable capacity strengthening

The current challenges for African doctoral training centres include limited capacity and the unstructured nature of the doctoral training programmes [2]. There has been increased interest in improving investments in doctoral education and expansion in PhD training in Africa in recent years, including through the World Bank [67]. Doctoral training is essential for the generation of new knowledge and sustainable development, including economic growth [1,2]. Efforts to position Africa as a giant for knowledge generation through doctoral training and research are currently underway [7,80]. Although this is likely to be critical for the socio-economic development of most countries, there are potential trade-offs in terms of quality versus quantity of research outputs. In view of this concern, PhD students need to be trained and nurtured to be critical thinkers and the creative problem solvers that society needs, not just specialists within a limited area of interest [63]. In their recent commentary, Hewitson [6] highlighted that addressing global scientific capacity imbalances through capacity building enables venturing into local technology to sustain infrastructure, which consequently allows developing countries to explore tailored solutions for local challenges. To ensure this, there is a need for long-term mentoring of trainees [6], in addition to external support to train doctoral students and build partnerships equitably with local collaborators rather than engaging in extractive research [74]. Follow-on postdoctoral or fellowship funding to build on the PhD training and availability of tenured academic positions is crucial for sustainable capacity strengthening and retention of researchers.

### 7.2. Wider institutional and social constraints to sustainable capacity building

The African Union’s Strategic Agenda 2063 seeks to accelerate the implementation of past and existing continental initiatives for growth and sustainable development [73], and this requires quality research institutions and mechanisms to support both students and academic supervisory/mentoring staff during PhD training. Many students who train abroad are often hesitant to return to local institutions because of the lack of opportunities there and political and economic instabilities. Effort needs therefore to be channelled towards incentivizing researchers to return and invest their knowledge towards the achievement of set strategic objectives and goals.

Enabling environments, confidence building and long-term mentoring of trainees is crucial to enable independent growth and long-lasting benefits to institutions and countries at large. However, experiential confidence is the most valuable capacity, upon which the other two can be strengthened [6]. Some of the institutional impediments that remain a major cause for concern include principal investigators who aim to grow their own personal interests, visibility and academic excellence at the expense of ensuring the visibility of trained students at the end of their PhD journey. On the other hand, doctoral students are expected to study full-time but often stipends in African institutions are not competitive. This is even though, in most cases, potential PhD candidates embark on their studies when they already have family responsibilities. The small allowances allocated to PhD students often lead to frustrations and resentment towards academia and research, and even mental health challenges.

Considerations for mental health should also be taken seriously. Over 30% of PhD students in Belgium, a developed country, were reported to be at risk of having or developing a common psychiatric disorder, especially depression [81]. This trend could potentially be reported in developing countries due to similarly tougher organizational policies, work–family trade-offs and perceptions of better career structures outside academia. Resources and training should thus be channelled towards equipping PhD students to deal with mental health issues which could potentially arise from organizational policies, work–family interface, job demands and job control, the supervisor’s leadership style, team decision-making culture and perception of a career outside academia [81]. This might be costlier to the funders but fundamental to the students who are an important investment towards developing more sustainable research and development activities in SSA.

## 8. Summary and conclusions

Developmental efforts within the research sector in Africa continue to face various challenges. Programmes such as the RS–FCDO ACBI are vital for research capacity building and supporting the growth of the knowledge economy in Africa, which is important in addressing current societal and environmental constraints. Learnings from the Soil Geochemistry consortium within the ACBI initiative is an example of research approaches for sustainable global, regional and national research partnerships. Interactions between the consortia and non-consortia colleagues within the partner institutions and additional funding pursued as appropriate, ensures ownership and continuity of research work, which often requires implementation post-award of grants. The embedment of a technical capacity-strengthening theme within the Soil Geochemistry consortium, in addition to scientific strengthening and doctoral training is important for long-term research sustainability.

The projected demographic dividend changes present opportunities for socio-economic transformation in SSA, but this requires investment and a shift in thinking from traditional research managed by external factors. Strengthening research capacity in SSA through doctoral training and establishment of sustainable multidisciplinary scientific networks can contribute to more self-reliant and sustainable research in the future.

## Data Availability

The top 50 keywords/phrases by relevance, based on 21 publications from the consortium were generated by SciVal. All data presented in the manuscript have been published in peer-reviewed journals. Sources of data are cited throughout the manuscript. No new additional data were generated and presented in this manuscript.
